# Activation of P2X7 Receptors in Peritoneal and Meningeal Mast Cells Detected by Uptake of Organic Dyes: Possible Purinergic Triggers of Neuroinflammation in Meninges

**DOI:** 10.3389/fncel.2019.00045

**Published:** 2019-02-13

**Authors:** Dilyara Nurkhametova, Igor Kudryavtsev, Valeriia Guselnikova, Maria Serebryakova, Raisa R. Giniatullina, Sara Wojciechowski, Fatma Tore, Albert Rizvanov, Jari Koistinaho, Tarja Malm, Rashid Giniatullin

**Affiliations:** ^1^Laboratory of Neurobiology, Kazan Federal University, Kazan, Russia; ^2^A.I.Virtanen Institute for Molecular Sciences, University of Eastern Finland, Kuopio, Finland; ^3^Department of Immunology, Institute of Experimental Medicine, St. Petersburg, Russia; ^4^Department of Fundamental Medicine, Far Eastern Federal University, Vladivostok, Russia; ^5^Department of General and Special Morphology, Institute of Experimental Medicine, St. Petersburg, Russia; ^6^School of Medicine, Biruni University, Istanbul, Turkey; ^7^Department of Exploratory Research, Scientific and Educational Center of Pharmaceutics, Kazan Federal University, Kazan, Russia; ^8^Neuroscience Center, Helsinki Institute of Life Science, University of Helsinki, Helsinki, Finland

**Keywords:** mast cells, ATP, P2X7 receptor, degranulation, neuroinflammation, migraine

## Abstract

Extracellular ATP activates inflammasome and triggers the release of multiple cytokines in various immune cells, a process primarily mediated by P2X7 receptors. However, the expression and functional properties of P2X7 receptors in native mast cells in tissues such as meninges where migraine pain originates from have not been explored. Here we report a novel model of murine cultured meningeal mast cells and using these, as well as easily accessible peritoneal mast cells, studied the mechanisms of ATP-mediated mast cell activation. We show that ATP induced a time and dose-dependent activation of peritoneal mast cells as analyzed by the uptake of organic dye YO-PRO1 as well as 4,6-diamidino-2-phenylindole (DAPI). Both YO-PRO1 and DAPI uptake in mast cells was mediated by the P2X7 subtype of ATP receptors as demonstrated by the inhibitory effect of P2X7 antagonist A839977. Consistent with this, significant YO-PRO1 uptake was promoted by the P2X7 agonist 2′,3′-O-(benzoyl-4-benzoyl)-ATP (BzATP). Extracellular ATP-induced degranulation of native and cultured meningeal mast cells was shown with Toluidine Blue staining. Taken together, these data demonstrate the important contribution of P2X7 receptors to ATP-driven activation of mast cells, suggesting these purinergic mechanisms as potential triggers of neuroinflammation and pain sensitization in migraine.

## Introduction

Mast cells are well-known players in allergic responses and essential contributors to inflammation in various tissues (Galli and Tsai, 2012). When activated, mast cells release multiple substances such as biogenic amines, histamine and serotonin, enzymes β-hexosaminidase, chymase and tryptase, and a number of pro-inflammatory cytokines and growth factors (Wernersson and Pejler, 2014). The particular profile of these secreted agents determines the type of inflammatory responses in surrounding tissues. Notably, mast cells are tissue resident and their morphology and functional role are tissue-dependent according to the local microenvironment and triggering stimuli (Galli et al., 2011). Therefore, the data obtained from one population of mast cells cannot be simply extrapolated to another type of mast cells.

Recently, much attention has been paid to the role of meningeal mast cells as the triggering actor in migraine attack. It has been suggested that degranulation of mast cells located in meningeal tissues contributes to pain signaling in migraine (Levy, 2009, 2012; Kilinc et al., 2017). However, the main missing piece of information in this hypothesis concerns the nature of the endogenous trigger for degranulation of mast cells in meninges *in situ*.

A purinergic hypothesis of migraine, originally proposed by Burnstock (1981), was complimented by a more recent hypothesis suggesting the role of ATP-gated P2X3 receptors in generation of migraine pain (Giniatullin et al., 2008; Yegutkin et al., 2016; Zakharov et al., 2016). However, given the presence of multiple types of ATP receptors in meningeal tissues, the full spectrum of ATP driven mechanisms in migraine remains incomplete. For instance, it is well established that extracellular ATP activates ligand-gated P2X7 receptors present in the majority of immune cells thus leading to a release of multiple pro-inflammatory cytokines and activation of inflammasome (Sperlágh and Illes, 2014; Franceschini et al., 2015; Burnstock, 2016; Karmakar et al., 2016). Consistent with this, P2X7 receptor knockout animals have a blunted inflammatory response and failed to develop certain types of pain (Chessell et al., 2005). In contrast to other types of immune cells, the role of P2X7 receptors in mast cells is little explored. Nevertheless, there is evidence for the role of ATP-mediated mast cell dependent inflammation through P2X7 receptors in the intestine (Kurashima et al., 2012). P2X7 receptors are also characterized in human LAD2 mast cells, derived from a patient with mast cell leukemia (Wareham and Seward, 2016). However, there is still lack of information regarding the role of P2X7 receptors in activation of meningeal mast cells, which are potential players in migraine.

In this study, we developed a new model of cultured meningeal mast cells, and using a combination of various techniques, including time-lapse flow cytometry measurements, we show that mast cells obtained from the peritoneal cavity and from meninges, express ATP-gated P2X7 receptors and are permeable to organic molecules. ATP mediated degranulation of meningeal mast cells may be responsible for the activation of trigeminal nerve fibers and local neuroinflammation in the trigeminovascular system associated with migraine attack.

## Materials and Methods

### Animals

Experiments were performed on 10-12 week-old male C57BL mice obtained from the Animal Facilities of the University of Eastern Finland (UEF). The animal treatment procedures were approved by the Committee for the Welfare of Laboratory Animals of the University of Eastern Finland and the Provincial Government of Kuopio. All experiments were conducted in accordance with the guidelines of the European Community Council (Directives 86/609/EEC). All efforts were made to minimize the number of animals used and their suffering.

### Isolation and Identification of Mast Cells

To obtain meningeal mast cells, we adapted the method of dural immune cell isolation described by McIlvried et al. (2015). Animals under deep Avertin (tribromoethanol) anesthesia were perfused through the ascending aorta with phosphate buffer saline (PBS), pH 7.2. After decapitation, the head was cut along the sagittal suture and the brain was gently removed from hemispheres leaving intact meninges. For meningeal mast cell isolation, hemiskulls were gently scraped with pestles into PBS. The obtained cell suspension was transferred to ice-cold PBS supplied with 2% of heat inactivated fetal bovine serum (FBS) and centrifuged at 300 *g* for 5 min at 4°C. The pellet was resuspended in PBS, filtered through 70 μm pre-separation filters (Miltenyi Biotec, Germany) and used for mast cell identification.

Peritoneal mast cells were isolated as described previously by Jensen et al. (2006) with slight modifications to improve cell viability and minimize baseline mast cell activation: lavage procedure was performed using ice-cold PBS with 2% FBS and all following steps were conducted at 4°C. The obtained pellet was resuspended in PBS and filtered through 50 μm filters (Sysmex CellTrics^®^, Germany).

For flow cytometry characterization, peritoneal or meningeal cells were stained with anti-mouse FcεRI conjugated with Alexa Fluor^®^ 647 (clone MAR-1, BioLegend, USA), and CD117 conjugated with tandem dye APC/Cy7 (clone 2B8, Biolegend) antibodies for 15 min at room temperature, washed with PBS with 2% FBS (300 g for 5 min) and resuspended in 300 μl of fresh PBS. Cell viability was determined using SYTO 16 Green Fluorescent Nucleic Acid Stain (Thermo Fisher Scientific, Waltham, MA, USA).

The data were acquired using BD FACSAria™ III cell sorter (BD Biosciences, San Jose, CA, USA) equipped with 488 and 633 nm lasers. SYTO 16 is excited by the 488 nm laser and detected through 530/30 filter. Phenotyping marker fluorochromes are excited by the 633 nm laser and detected through 660/20 and 780/60 filters for Alexa Fluor^®^ 647 and APC/Cy7, respectively. Compensation for the spillover of fluorochromes into other channels was made using single stained cells.

### Culturing of Peritoneal and Meningeal Mast Cells

Unfractionated peritoneal cells or cells obtained by hemiskull scraping were centrifuged at 300 *g* for 5 min at 4°C. The pellet was re-suspended in Dulbecco’s Modified Eagle Medium (DMEM) supplemented with 10% FBS, 1% antibiotics (penicillin/streptomycin), 2 mM L-glutamine, 50 μM B-mercaptoethanol, 10 ng/ml murine recombinant stem cell factor (SCF; PeproTech, NJ, USA), and 10 ng/ml murine recombinant interleukin (IL)-3 (PeproTech, NJ, USA). After 2–3 weeks of culture, more than 98% of cells were identified as mast cells by Toluidine Blue staining. Cells were kept in culture for up to 5 weeks.

### Toluidine Blue Staining of Meningeal Mast Cells

Whole mount meninges on hemiskulls were pre-treated with or without 1 mM ATP in artificial cerebrospinal fluid (ACSF) containing (in mM): NaCl 115, KCl 3, CaCl_2_ 2, MgCl_2_ 1, NaH_2_PO_4_ 1, NaHCO_3_ 25 and glucose 11; bubbled with 95% O_2_/ 5% CO_2_) for 10 min at room temperature. Then samples were fixed with 4% paraformaldehyde at 4°C overnight. After rinsing with PBS, meninges were carefully dissected from the skull, and put on a glass coated with poly-L-lysine (Polysine^®^ Thermo-Scientific, USA). Staining with Toluidine Blue (pH 2.0) was performed according to the standard protocol we described previously (Levy et al., 2007; Kilinc et al., 2017). Images were captured using Olympus AX-TFSM microscope (Olympus, Japan). The number of granulated and degranulated mast cells in each meninges (*n* = 5) was counted in five random areas containing the main branches of the middle meningeal artery by an observer blinded to treatment groups. Mast cells were classified as degranulated if they were pale, poorly stained, had distorted cytoplasmic boundaries, and surrounding positively stained granules (Shelukhina et al., 2017).

### Stimulation of Peritoneal and Meningeal Mast Cells With ATP

To study P2X7 receptor activation in freshly isolated peritoneal and meningeal mast cells, the cells were treated with different concentrations of ATP and 2′,3′-O-(benzoyl-4-benzoyl)-ATP (BzATP; both from Sigma-Aldrich, Germany). Notably, BzATP is more potent than ATP as an agonist at P2X7 receptors whereas it is equally or less potent than ATP at other P2X receptors (North and Surprenant, 2000).

ATP-induced mast cell activation was evaluated using the fluorescent dye YO-PRO1 (Thermo Fisher Scientific, Waltham, MA, USA) which enters the cells through the dilated P2X7 receptor ion channel (Michel et al., 1999; Browne and North, 2013; Browne et al., 2013). ATP at final concentrations 100 μM, 1 mM or 5 mM or BzATP to a final concentration of 100 μM were added and samples were incubated for 20 min in the dark at room temperature, followed by addition of 1 μM of YO-PRO1. After incubation, 200 μl of fresh PBS was added.

Samples were run on a BD FACSAria III cell sorter (BD Bioscience). YO-PRO1 is excited by the 488 nm laser and detected through 530/30 filter. The data were shown as a percentage of YO-PRO1 positive cells in each sample as previously reported (Karmakar et al., 2016).

Cultured mast cells, before stimulation, were washed once with Dulbecco’s PBS, and then centrifuged at 300 g for 5 min at 4°C, and the pellet was resuspended in 1 ml of PBS. A cell suspension (5 × 10^5^ cells/ml) was plated onto 24-well plates (100 μl per well). Meningeal cell-derived mast cells (MDMCs) were stimulated with ATP at final concentration 1 mM for 5 min at room temperature. Peritoneum-derived mast cells were stimulated with ATP (100 μM, 1 or 5 mM) or BzATP (100 μM) at room temperature. For inhibitory experiments, peritoneum-derived mast cells were pre-treated with P2X7 antagonist A839977 (10 μM) for 5 min followed by 1 mM ATP stimulation. Application of PBS was used as the control. After incubation, mast cells were transferred onto glass microscope slides, dried at 37°C, and stained with Toluidine Blue. The number of intact and degranulated mast cells was counted randomly and blindly in five fields on each slide. Mast cells were defined as stated above (Shelukhina et al., 2017).

### Time-Lapse Analysis of DAPI Fluorescence

It has been recently shown that P2X7 receptors are also permeable to the DNA dye 4,6-diamidino-2-phenylindole (DAPI; Bukhari et al., 2016). We used flow cytometry to determine the time-course of DAPI uptake (excitation/emission 405/450 nm) by mast cells. Peritoneal mast cells were identified based on FcεRI and CD117 expression as described above. Samples were analyzed using the Cytoflex flow cytometer equipped with 405, 488, and 638 nm lasers (Beckman Coulter Inc., CA, USA). A peristaltic pump in this device allowed the addition of the agonist ATP during on-line acquisition of data. ATP at final concentrations 100 μM or 1 mM was added at 20 s after the beginning of the recording. Up to 25,000 peritoneal mast cells per sample were acquired during 120 s. All flow cytometric data were analyzed using CytExpert Software v 1.3 or Kaluza Software v 1.5 (Beckman Coulter Inc., CA, USA). DAPI (1 μg/ml) incorporation was measured by using median fluorescence intensity (MFI) of single cells after ATP application or control. The data from independent experiments were tested for normality of distribution by the Kolmogorov-Smirnov test (*n* > 50) at each time point.

### Statistical Analysis

Data were analyzed using Statistica 8 Software (Quest Software Inc., Aliso Viejo, CA, USA), Origin (Origin labs, MS, USA) and GraphPad Prism 4 (GraphPad Software, La Jolla, CA, USA). Statistical analysis was performed using nonparametric Mann-Whitney *U* test, Student *t*-test or one-way ANOVA, followed by Dunnett’s multiple comparisons test when appropriate. Differences with *p* values of less than 0.05 were considered statistically significant. The data are presented as mean ± SEM.

The raw data supporting the conclusions of this manuscript will be made available by the authors, without undue reservation, to any qualified researcher.

## Results

### Identification of Mast Cells

**Figure 1:**
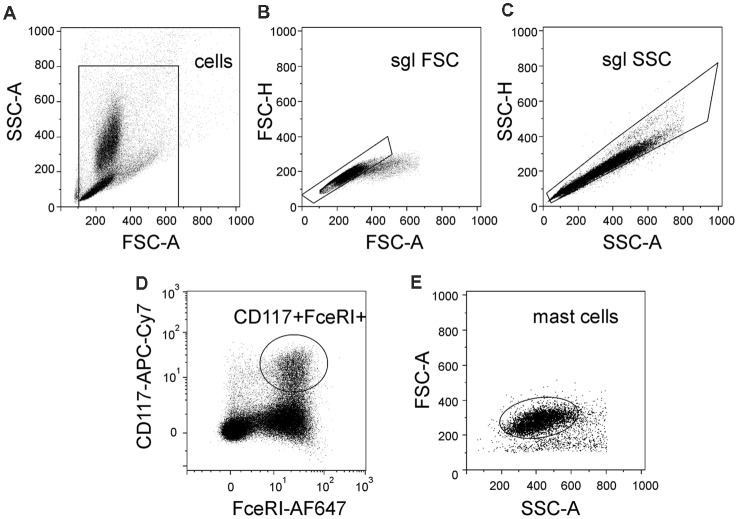
Flow cytometric gating strategy used to identify mast cells. **(A)** Light scatter profile for cells based on forward scatter (FSC-A) and side scatter (SSC-A) (the region is set to discriminate between cells and debris). **(B,C)** Singlet gating based on FSC-H vs. FSC-A and then SSC-A vs. SSC-H, respectively (the regions are set to discriminate cell doublets). **(D)** Cells were divided into subpopulations based on Alexa Fluor 647-FcεRI and APC-Cy7-CD117 (the region is set to discriminate between double-positive cells and all other subpopulation of cells). **(E)** Back-gating of double positive cells onto the light scatter plot FSC-A vs. SSC-A. This shows relative size and granularity of mast cells compared to those in **(A)**. Cells from the region of “mast cells” were used further to assess the action of ATP on mast cell functionality.

In order to distinguish a population of murine mast cells from other cell types localized in meninges or in the peritoneal cavity, the cell surface expression of FcεRI and CD117 was determined. FcεRI is a mast cell membrane receptor specific for IgE, which is a potent inducer of mast cell activation and degranulation (Rivera and Gilfillan, 2006) whereas CD117 (also named c-kit) is a receptor for the SCF important for mast cell migration, survival and proliferation (Yamazaki et al., 2015). Figure 1 shows the gating strategy of our protocol to obtain the final fraction of mast cells. Thus, there are light scatter dot plots for isolated cells based on forward scatter (FSC-A) related to light refraction and cell size and side scatter (SSC-A) reflecting cell granularity (Figure 1A). The selected region from the light scatter plot, which eliminates debris, was set to remove doublet cell aggregates (Figures 1B,C). Cells were further divided into subpopulations based on expression of FcεRI and CD117 (Figures 1D,E) to isolate the fraction of “mast cells.” Cells with double positive expression of both FcεRI and CD117 were identified as mast cells. This approach allowed us to identify a pure fraction of mast cells, which were further tested with the purinergic agonists.

### ATP-gated P2X7 Receptor Mediates Peritoneal Mouse Mast Cell Degranulation

**Figure 2:**
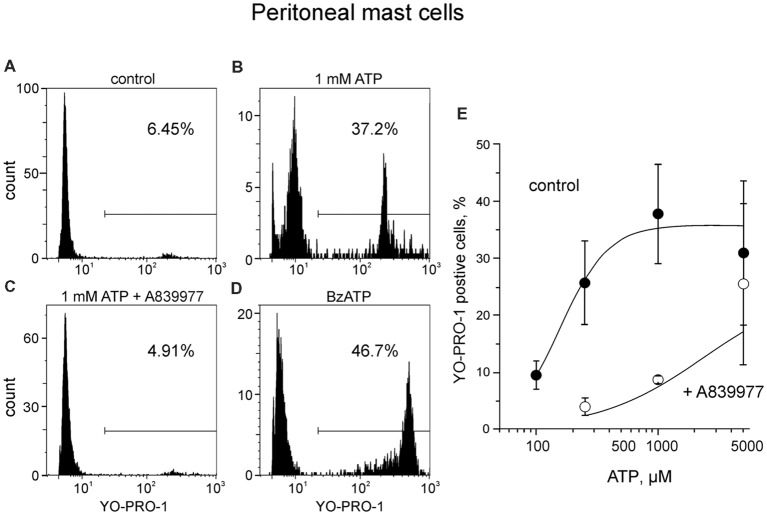
ATP induces the uptake of YO-PRO1 by peritoneal mast cells. **(A–D)** Representative histograms (fluorescence intensity vs. number of cells) of YO-PRO1 incorporation into murine peritoneal mast cells. **(A)** Negative control of untreated murine peritoneal mast cells incubated with 1 μM of YO-PRO1 for 15 min (*n* = 11). **(B)** Mast cells pre-treated with 1 mM of ATP for 15 min and stained with 1 μM of YO-PRO1 (*n* = 8, *p* < 0.01). **(C)** Mast cells in the presence of P2X7 antagonist A839977 (5 μM), then stimulated with 1 mM of ATP for 15 min and stained with 1 μM of YO-PRO1 (*n* = 4). **(D)** Mast cells pre-treated with 100 μM of 2′,3′-O-(benzoyl-4-benzoyl)-ATP (BzATP) for 15 min and stained with 1 μM of YO-PRO1 (*n* = 5, *p* < 0.05). **(E)** The dose-response curve showing percent of mast cells which incorporated YO-PRO1 after stimulation with increasing doses of ATP and in the presence of the P2X7 antagonist A839977 (5 μM). Mean ± SEM (one-way ANOVA, followed by Dunnett’s multiple comparison test).

First, to optimize our technical approach, we evaluated the ability of ATP to activate ATP-gated P2X7 receptors in easily accessible peritoneal mouse mast cells by using YO-PRO1 which is able to penetrate the cell membrane during the activation of P2X7 receptors (Browne et al., 2013). As expected, unstimulated mast cells failed to take up YO-PRO1, as only 8.2 ± 1.3% (*n* = 11) of the cells contained YO-PRO1 (Figure 2A). Application of 1 mM ATP for 15 min increased the uptake up to 37.9 ± 9.2% (*n* = 8, *p* < 0.01; Figure 2B). P2X7 specific antagonist A839977 (Honore et al., 2009) prevented the ATP induced increase in YO-PRO1 signal (8.7 ± 0.6%, *n* = 4; Figure 2C) whereas application of P2X7 agonist BzATP (Bianchi et al., 1999) effectively enhanced the YO-PRO1 loading of the cells by 32.2 ± 10.9% (*n* = 5, *p* < 0.05; Figure 2D). Next, we demonstrated the dose-dependent action of ATP on YO-PRO1 uptake (Figure 2E). Stimulation of mast cells with increasing concentrations of ATP led to increased YO-PRO1 uptake. Pre-treatment with P2X7 antagonist A839977 (5 μM) prevented the ATP-induced YO-PRO1 incorporation (Figure 2E).

**Figure 3:**
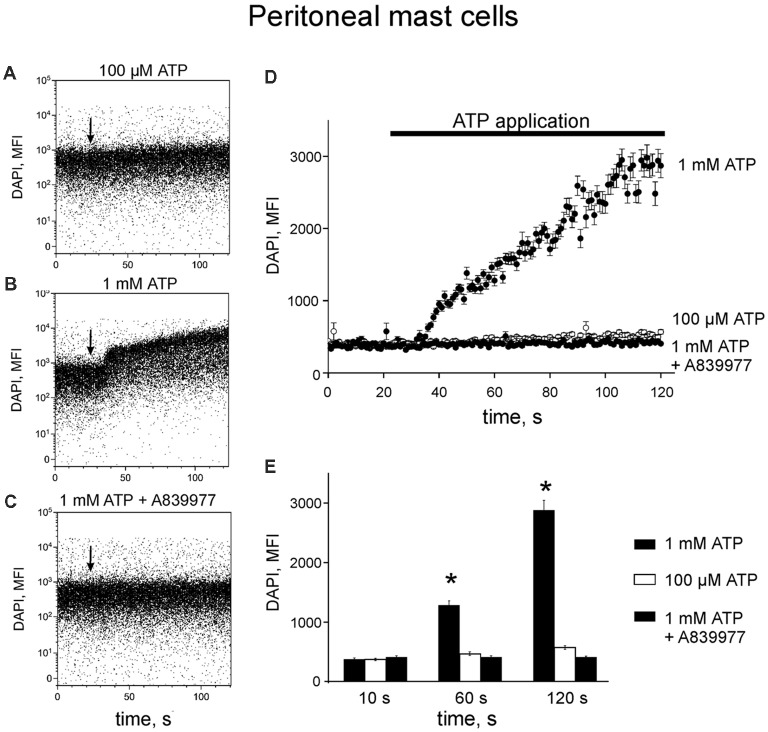
ATP induces 4,6-diamidino-2-phenylindole (DAPI) uptake by peritoneal mast cells. **(A–C)** Representative dot-plots [time vs. DAPI (1 μg/ml) fluorescence intensity] obtained from murine peritoneal mast cells stimulated with 100 μM and 1 mM ATP, 1 mM ATP in the presence of 150 nM P2X7 antagonist A839977 (dot-plots **A**, **B** and **C**, respectively). The X-axis represents the time (seconds) from the beginning of sample acquisition, ATP was added at 20 s; the Y-axis represents the relative fluorescence of DAPI (notice log scale). **(D)** Pooled DAPI fluorescence intensity data for murine peritoneal mast cells (*n* = 3) stimulated with 100 μM and 1 mM ATP, and 1 mM ATP + 150 nM P2X7 antagonist A839977, respectively. **(E)** Histograms showing DAPI fluorescence at three different time points before and after application of 100 μM ATP (white), 1 mM ATP (black) alone or 1 mM ATP in the presence of 150 nM P2X7 antagonist A839977 (gray). Mean ± SEM, *n* = 3, *p = 0.049 (paired sample Student *t*-test).

Next, we reconstructed a time-course for ATP-induced responses of P2X7 allowing us to characterize the early events in the activation of P2X7 receptors. Application of 100 μM ATP on murine peritoneal mast cells induced a slight increase in DAPI fluorescence by 120 s (Figures 3A,D,E) whereas in samples treated with 1 mM ATP, a robust enhancement of DAPI fluorescence was observed during the 120 s recording time (Figures 3B,D,E). Specific P2X7 receptor antagonists prevented the stimulatory effect of 1 mM ATP on DAPI fluorescence (Figures 3C–E).

**Figure 4:**
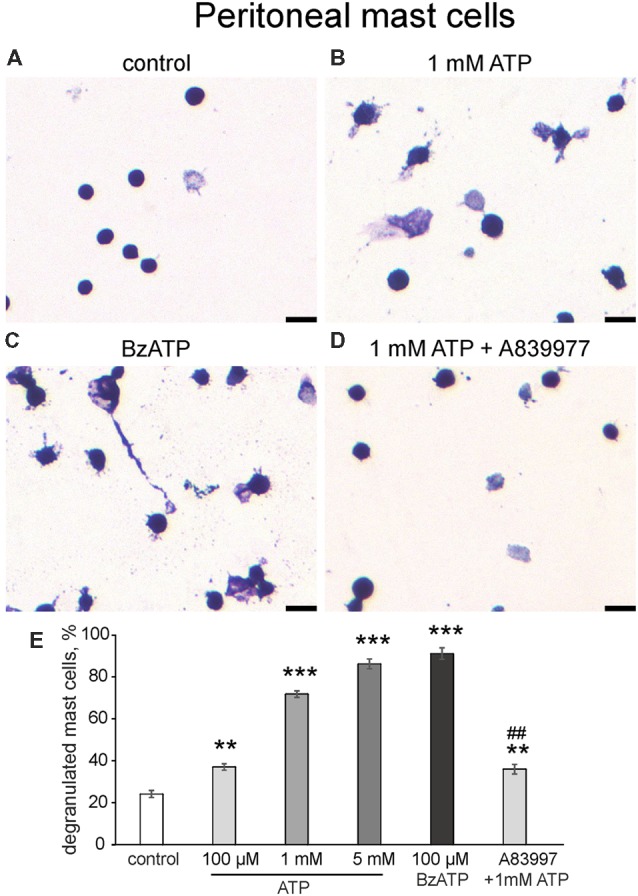
Degranulation of cultured peritoneal mast cells induced by ATP and BzATP. **(A–D)** Toluidine Blue staining of cultured peritoneal mast cells (20×). **(A)** Control conditions, **(B)** application of 1 mM ATP, **(C)** application of 100 μM BzATP, **(D)** pre-treatment with P2X7 antagonist A839977 (10 μM) followed by 1 mM ATP stimulation. **(E)** Histograms showing a percent of degranulated mast cells in control, after application of ATP (100 μM, 1 mM, 5 mM) and BzATP (100 μM), and after pre-treatment with A839977 (10 μM) followed by application of 1 mM ATP. Calibration bar 100 μm. Mean ± SEM, *n* = 4, **p ≤ 0.005 vs. control, ***p < 0.0001 vs. control, ^##^*p* < 0.0001 vs. 1 mM ATP group (one-way ANOVA, followed by Dunnett’s multiple comparison test).

In addition to the flow cytometry approach, we confirmed the ATP action on cultured peritoneal mast cells by morphological analysis (Figure 4). In control conditions, most mast cells were intact, and exhibited dense, compact, unbroken cytoplasmic boundaries and did not have many surrounding granules (Figure 4A). Application of 1 mM ATP (Figure 4B) or 100 μM BzATP (Figure 4C) increased the number of mast cells with blurred contours and numerous granules around the cells which is indicative of degranulation. Notably, even 100 μM ATP significantly increased mast cell degranulation (by 37.1 ± 1.5%, *n* = 4, *p* = 0.0013). However, a much higher level of degranulation was observed with 1 mM and 5 mM ATP. Consistent with this the P2X7 agonist BzATP (100 μM) effectively degranulated most mast cells. Pre-treatment with the P2X7 antagonist A839977 (10 μM) suppressed the ATP-induced degranulation (Figure 4D). Thus, consistent with flow cytometry data, two agonists (ATP and BzATP) induced significant degranulation of peritoneal mast cells mediated by the P2X7 receptors.

### Long-Term Culturing Enriched Mature Meningeal Mast Cells

**Figure 5:**
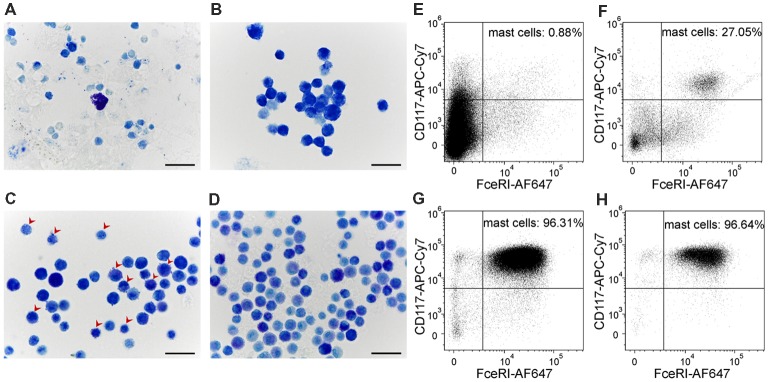
Cultured meningeal mast cells exhibit typical mast cell morphology. **(A–D)** Toluidine Blue staining of **(A)** freshly isolated meningeal mast cells, **(B)** cells kept in culture for 1 week, **(C)** 2 weeks (red arrows showing the presence of both blue and violet granules in the cytoplasm of mast cells), and** (D)** 3 weeks. **(E–H)** Cultured mast cells were identified by their surface expression of FcεRI and CD117. The percentage of FcεRI+CD117+ cells increased upon time in culture. **(E)** freshly isolated cells, **(F)** cells after 1 week in culture, **(G)** cells after 2 weeks in culture, **(H)** cells after 3 weeks in culture.

In order to evaluate P2X7-receptor activation on a more relevant model of mast cells, we developed a method to culture mouse meningeal mast cells. Freshly isolated meningeal cells were identified in Toluidine Blue stained slides by their rounded shape and average size of 12 μm (Figure 5A). The granules of meningeal mast cells were always stained metachromatically in violet by Toluidine Blue. All other meningeal cells, their nuclei and the nuclei of mast cells were stained orthochromatically in blue (Figure 5A). During 1 month of observations, the cultures were significantly enriched by mast cells (Figures 5A–D). After 1 week in culture, all cells showed similar morphological features: a rounded shape with an average size of 7.95 ± 1.1 μm and poorly visualized granules in the cytoplasm. The cytoplasm was stained in tones from light blue to blue in the presence of Toluidine Blue (Figure 5B). After 2 weeks of cultivation, the cultured cells retained a round shape and had an average size of 7.33 ± 0.96 μm. After two and three weeks, granules were seen within the cytoplasm of these cells (Figure 5C, red arrows). After 3 weeks of cultivation, cells exhibited a rounded shape and the cytoplasm was filled with metachromatically (violet) stained granules (Figure 5D). Mature mast cells obtained from the culture were heterogeneous in size (average cell size was 7.3 ± 0.97 μm) and density of metachromatic granules. The MDMCs maintained such morphology up to 3 months of culture.

Murine mast cells were identified from other cell types localized in meninges or in the peritoneal cavity based on their cell surface expression of FcεRI and CD117 (Figures 5E–H). Figure 1 shows the gating strategy to identify a pure fraction of mast cells. Less than 1% of the freshly isolated cells from meningeal tissues were FcεRI+CD117+ positive. By the end of the first week of culturing the percentage of FcεRI+CD117+ cells increased up to 27%, reaching over 95% by the second week in culture (Figures 5E–H). The percentage of viable FcεRI+CD117+ mast cells remained over 95% up to fifth week of culture. The culture viability remained between 81% and 100% for the first 1–4 weeks and decreased to 62.4% during the fifth week in culture (data not shown).

### P2X7 Receptors Are Expressed in Meningeal Mouse Mast Cells

**Figure 6:**
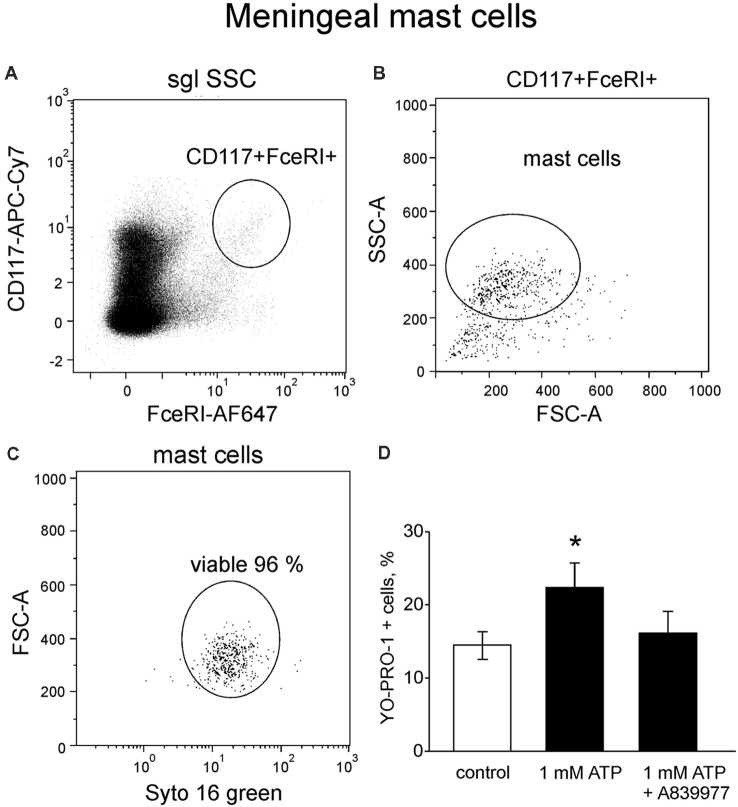
YO-PRO1 uptake in meningeal mast cells in response to ATP. **(A)** Flow cytometric gating strategy used to identify mouse meningeal mast cells is first based on CD117 and FcεRI labeling. **(B)** Next elimination of debris by gating of mast cells based on light scattering properties FSC and SSC. **(C)** Viable meningeal mast cells are gated on the viability dye SYTO 16 Green Fluorescent Nucleic Acid Stain. **(D)** Stimulation with ATP led to increase of YO-PRO1 positive cells (*n* = 8) whereas pre-treatment with the P2X7 antagonist A839977 inhibited incorporation of YO-PRO1 (*n* = 5). Mean ± SEM, (*n* = 10 in control), *p = 0.016 (Mann-Whitney *U* test).

Next, in order to investigate the tissue specific properties of mast cells in the dura mater, where they are likely to be involved in triggering of migraine attack (Levy, 2009; Kilinc et al., 2017), we explored P2X7 receptor activation in meningeal mast cells. Mouse meningeal mast cells were identified based on labeling with CD117 and FcεRI (Figure 6A) and further separated from debris based on their light scatter characteristics (FCS-A vs. SSC-A, Figure 6B). The final population contained at least 95% of viable meningeal mast cells (Figure 6C) which were tested for ATP-induced P2X7 activation. In control conditions, in a population of freshly purified cells obtained from meninges, the percentage of YO-PRO1 uptake was low, approximately 14.4 ± 1.9% (Figure 6D). Incubation with 1 mM ATP significantly enhanced the uptake of the dye (Figure 6D). Similar to the peritoneal cells, the treatment with the P2X7 antagonist A839977 (5 μM) prevented the ATP induced YO-PRO1 uptake (Figure 6D).

**Figure 7:**
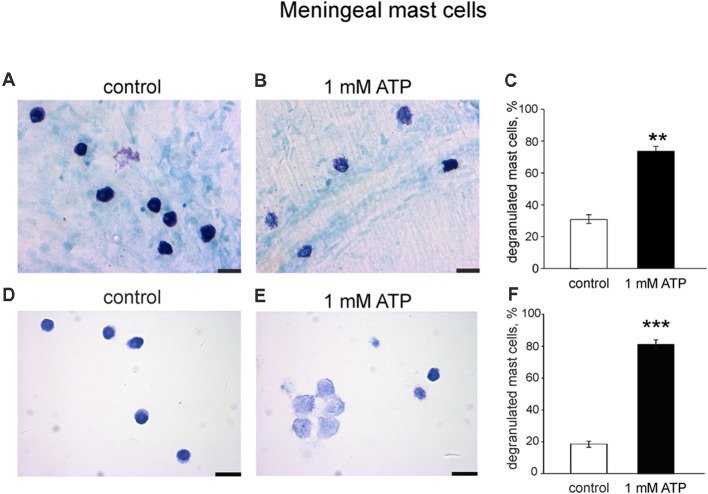
Degranulation of native and cultured meningeal mast cells with ATP. **(A,B)** Toluidine Blue staining of mast cells in whole mount meninges (20×). Mast cells are mostly intact in control conditions **(A)**. After treatment with 1 mM ATP the number of degranulated mast cells significantly increased **(B)**. **(C)** Histograms showing a fraction of degranulated cells in control and after application of 1 mM ATP. Calibration bar 20 μm. Mean ± SEM, *n* = 5, **p = 0.008. **(D,E)** Toluidine Blue staining of cultured mast cells (40×). **(D)** Control conditions, **(E)**, application of 1 mM ATP. **(F)** Histograms showing a percentage of degranulated mast cells in control and after application of 1 mM ATP. Calibration bar 50 μm. Mean ± SEM, *n* = 4, ***p < 0.0001 vs. control (Mann-Whitney *U* test).

To explore if the purinergic challenge has a functional impact on the release of active components from granules we tested the degranulation ability of 1 mM ATP on mouse meningeal mast cells in whole mount meningeal tissues as identified by Toluidine Blue staining (Levy et al., 2007; Kilinc et al., 2017). In naïve isolated meninges most mast cells were intact (Figure 7A), whereas ATP triggered degranulation of multiple mast cells localized near the meningeal artery (Figure 7B). Figure 7C shows pooled data obtained from five mice, indicating the ability of ATP to induce significant degranulation of mast cells in the dura mater. These findings were further confirmed in cultured meningeal mast cells by morphological analysis. In control conditions most mast cells were intact (Figure 7D), and the application of 1 mM ATP significantly increased the number of degranulated mast cells (Figures 7E,F).

## Discussion

Here, we show for the first time that rodent mast cells derived from meninges can be grown, matured and enriched in long-term culture. The ability of mast cell granules to show metachromasia, that is, to display a color different from that of the applied dye, is a key feature of mature mast cells. In mammalian mast cells, the distinctive property of metachromasia is accounted for the presence of heparin, a sulfur-rich glycosaminoglycan, in mast cell secretory granules (Härmä and Suomalainen, 1951). Based on these data, blue (orthochromatic) staining of mast cell granules, which was observed after 1 week of cultivation, may indicate immaturity. The appearance of violet (metachromatic) granules in the cell cytoplasm after 2 weeks of cultivation is evidence of active maturation. The presence of metachromatic granules in almost all cells after 3 weeks of cultivation indicates the functional maturity of the mast cells at this time point.

The high-affinity IgE receptor (FcεRI) and CD117 (c-Kit) we used for flow cytometry are classical mast cell markers. Both mast cell progenitors and mature mast cells express these cell surface markers (Dahlin et al., 2015). This explains the high percentage of double positive cells already after 2 weeks of cultivation when only a few cells were identified to contain metachromatic stained granules in the presence of Toluidine Blue. Comparing the obtained flow cytometry data with the Toluidine Blue staining data, it can be concluded that the MDMCs have reached maturity after 3 weeks of cultivation. At this time point, more than 95% of the living cells in culture express FcεRI and CD117 and contain metachromatically violet stained (mature) granules in the cytoplasm. Based on these results, we recommend the use of MDMC culture 3–5 weeks after the start of cultivation.

By using these and the easily accessible peritoneal mast cells, we characterized the role of ATP-gated P2X7 receptors associated with the uptake of organic dyes. The ability of ATP to degranulate meningeal mast cells suggests that this extracellular purinergic messenger could act as an endogenous trigger of neuroinflammation in various neurological disorders, including meningitis and migraine. ATP-gated P2X7 receptors are important triggers of neuroinflammation in different tissues. The activation of P2X7 receptors in mast cells is associated with release of pro-inflammatory cytokines such as IL-1β, IL-18 (Ferrari et al., 2006) and IL-6 (Shieh et al., 2014) which are essential contributors to neuropathic and inflammatory pain (Chessell et al., 2005; Sperlágh and Illes, 2014). The role of the NLRP3 inflammasome and release of IL-1β have been shown also in pneumococcal meningitis (Zwijnenburg et al., 2003) suggesting the involvement of P2X7 receptors in this pathology. However, recent testing with P2X antagonists did not reveal a significant change in the time-course of the disease which the authors explained by down-regulation of ATP receptors expression and decreased concentration of endogenous ATP (Zierhut et al., 2017). Unlike bacterial meningitis, in aseptic form of this disorder, so called drug-induced aseptic meningitis, headache is the leading symptom (Holle and Obermann, 2015), thus closely linking trigeminal pain and meningeal neuroinflammation.

P2X7 receptors are expressed in the majority of immune cells (Junger, 2011; Burnstock and Boeynaems, 2014). For instance, they have been found in macrophages (Moore and MacKenzie, 2007), monocytes (Humphreys and Dubyak, 1998; Grahames et al., 1999), neutrophils (Chen et al., 2004; Christenson et al., 2008), and different subtypes of T cells (Frascoli et al., 2012; Rissiek et al., 2015). However, P2X7 receptors are less studied in mast cells, which are often implicated in allergic reactions and in neuroinflammation. Nevertheless, one study reported that P2X7 receptors in mast cells play a role in gut inflammation (Kurashima et al., 2012). Another recent very detailed study, using a calcium imaging technique, demonstrated the functional expression of different P2X receptors, including P2X7 subtype, in human LAD2 mast cells (Wareham and Seward, 2016). The advantage of our study is that we focused on techniques, which allowed us to test the function of P2X7 receptors that is critical for initiation of neuroinflammation and compared two different populations of native mast cells.

Activation of P2X7 receptors in different cells is often followed by uptake of relatively large organic molecules such as the fluorescent dye YO-PRO1, which normally does not penetrate the cell membrane (Michel et al., 1999; Jindrichova et al., 2015; Bukhari et al., 2016). It is still a matter of debate whether these dyes penetrate the dilated ion channel of P2X7 receptor or enter through other P2X7 receptor associated proteins (Rassendren et al., 1997; Jiang et al., 2005; Pelegrin and Surprenant, 2006). Recent studies, however, showed that the P2X7 receptor permeability to organic cations such as YO-PRO1 is the intrinsic property of the ion channel itself determined by the long COOH-terminal tail reviewed recently by Di Virgilio et al. (2018). Thus, our data with the measurement of the fluorescence of YO-PRO1 and DAPI reflect, actually, the function of the ion channel of the P2X7 receptor opened by BzATP or ATP.

In the current project, using flow cytometry, we found that stimulation of mast cells with ATP led to P2X7 receptor mediated influx of YO-PRO1 in murine peritoneal and meningeal mast cells. This uptake was inhibited by the P2X7 antagonist A839977 suggesting either direct or indirect involvement of P2X7 receptors. A similar effect was observed using the P2X7 agonist BzATP and with relatively high concentrations of ATP. Taken together, these findings indicate a key role for P2X7 receptor in the activation of mast cells.

One novelty of our study was to use a flow cytometry technique to assess the permeability of mast cell membrane for the dye DAPI in real time after stimulation with 1 mM ATP. We found that stimulation with ATP caused DAPI influx into murine peritoneal mast cells in tens of seconds, and this effect was completely prevented by the P2X7 antagonist A839977. These data indicated the key role of P2X7 receptors in activation of mast cells.

It has been reported that human P2X7 receptor has essentially a higher affinity for several agonists than the mouse equivalent (Chessell et al., 1998). This suggests that the processes, which we observed in mouse cells, could be better presented in human tissues. Moreover, in humans, there are differences in dye uptake properties of the P2X7 receptor due to high polymorphism typical for this receptor type. Interestingly, this single nucleotide polymorphism can be linked (or probably even determine) lower pain sensitivity (Sorge et al., 2012). The latter observation, essential for personified pain medicine, highlights the need for further investigation of native P2X7 receptors in individual patients in order to evaluate the risk of pain state formation.

Mast cells are best known for their ability to release a plethora of various active substances. The early phase of mast cell activation leads to a release of pre-formed pro-inflammatory mediators from secretory granules followed by synthesis of lipid messengers, cytokines and chemokines (Boyce, 2005; Lorentz et al., 2012; Wernersson and Pejler, 2014). Classical mediators such as histamine and serotonin are released by different mechanisms such as degranulation (Dvorak, 1992; Moon et al., 2014) and constitutive or regulated exocytosis (Lacy and Stow, 2011; Lorentz et al., 2012; Moon et al., 2014). Degranulation of mast cells may be provoked by various stimuli such as antigens, monomeric IgE, neuropeptides (substance P, CGRP) and viruses involving different receptors and various signaling mechanisms (Moon et al., 2014). Among a number of stimuli, ATP emerged recently as an important trigger of mast cell activation (Wareham and Seward, 2016). These authors showed P2X7 mediated mast cell activation and degranulation in LAD2 mast cells by assessing calcium fluxes and β-hexosaminidase release (Wareham and Seward, 2016). In our study, we demonstrated not only that ATP activates native P2X7 receptors in meningeal mast cells but also showed that the application of ATP induces the release of granules from these cells.

According to common view, migraine pain is initiated by sensitized trigeminal nerve terminals in meninges within the so-called trigeminovascular system (Moskowitz, 1993; Levy, 2012; Zakharov et al., 2015). Meninges are occupied by a plethora of mast cells, which are localized at “strategic loci” close to main meningeal vessels and nerve fibers suggesting a functional interaction (Levy, 2009; Kilinc et al., 2017). Although we do not have direct evidence that mast cells degranulation causes activation of trigeminal nerve endings, there are data which provide evidence that activation of mast cells plays a triggering role in the underlying sensitization process (Levy et al., 2007; Kilinc et al., 2017). In a previous study, we developed the purinergic hypothesis of migraine originally proposed by Burnstock (1981), by showing that extracellular ATP activates primary afferents in meninges (Yegutkin et al., 2016). One open issue still remains: what is the source of extracellular ATP in migraine? There are plenty of potential sources to release ATP in the nervous system including astrocytes, neurons, platelets, and endothelial cells (Pangršič et al., 2007; Burnstock and Ralevic, 2014). In meninges, the main sources of ATP could be vessels, nerves and mast cells themselves. ATP-driven degranulation of mast cells is likely happening in migraine with aura since the cortical spreading depression is itself an inducer of meningeal mast cells degranulation and opening of pannexin1 channels (Karatas et al., 2013), which are permeable to ATP (Dahl, 2015). Notably, there is a positive feedback loop providing ATP-induced ATP release *via* pannexins (Dahl, 2015). This loop can amplify the initial signal to provide a level of extracellular ATP high enough to activate P2X7 receptors. A recent study indicated that the complex of P2X7 receptors and pannexins determines not only neuroinflammation but also the development of the cortical spreading depolarization, which is a key process underlying migraine aura (Chen et al., 2017).

We propose that the released extracellular ATP acts through the P2X7 subtype of purinergic receptors thus leading to both mast cells’ activation and degranulation. The main actor after degranulation of meningeal mast cells appears to be serotonin robustly exciting nerve terminals *via* ligand gated 5-HT3 receptors (Kilinc et al., 2017), whereas ATP can also act directly on nerve terminals *via* P2X3 receptors (Yegutkin et al., 2016; Zakharov et al., 2016). Taken together, these mechanisms contribute both to meningeal neuroinflammation and lasting pain formation in migraine.

In conclusion, we show the leading role of ATP-gated P2X7 receptors in activation and degranulation of mast cells that naturally reside in two different body compartments. Given the emerging appreciation of the role of mast cells in neuroinflammation, the present data could help to identify new therapeutic strategies to alleviate peripheral and central neurological disorders, including migraine.

## Author Contributions

DN, RRG and VG contributed to the data collection, analysis, interpretation and the manuscript writing. MS contributed to the data collection and analysis. SW contributed to the data collection and the manuscript editing. FT, AR and JK supervised the study. IK contributed to the study design, data collection, analysis, interpretation and the manuscript writing. TM and RG contributed to the study design and supervision, manuscript writing and final editing.

## Conflict of Interest Statement

The authors declare that the research was conducted in the absence of any commercial or financial relationships that could be construed as a potential conflict of interest.
